# Plasma Citrate Levels Are Associated with an Increased Risk of Cardiovascular Mortality in Patients with Type 2 Diabetes (Zodiac-64)

**DOI:** 10.3390/jcm12206670

**Published:** 2023-10-22

**Authors:** Arno R. Bourgonje, Margery A. Connelly, Harry van Goor, Peter R. van Dijk, Robin P. F. Dullaart

**Affiliations:** 1 Department of Gastroenterology and Hepatology, University of Groningen, University Medical Center Groningen, 9713 GZ Groningen, The Netherlands; 2 The Henry D. Janowitz Division of Gastroenterology, Department of Medicine, Icahn School of Medicine at Mount Sinai, New York, NY 10029, USA; 3 Labcorp, Morrisville, NC 27560, USA; connem5@labcorp.com; 4 Department of Pathology and Medical Biology, University of Groningen, University Medical Center Groningen, 9713 GZ Groningen, The Netherlands; h.van.goor@umcg.nl; 5 Department of Internal Medicine, Division of Endocrinology, University of Groningen, University Medical Center Groningen, 9713 GZ Groningen, The Netherlands; p.r.van.dijk@umcg.nl (P.R.v.D.); dull.fam@12move.nl (R.P.F.D.)

**Keywords:** type 2 diabetes, plasma citrate, cardiovascular mortality, macrovascular complications

## Abstract

Circulating citrate may represent a proxy of mitochondrial dysfunction which plays a role in the development of vascular complications in type 2 diabetes (T2D). Here, we determined the associations between plasma citrate levels and cardiovascular (CV) mortality in T2D patients. In this prospective cohort study, 601 patients were included who participated in the Zwolle Outpatient Diabetes project Integrating Available Care (ZODIAC). Plasma citrate levels were measured by nuclear magnetic resonance spectroscopy. Cox proportional hazards regression models were used to evaluate the associations between plasma citrate and the risk of CV mortality. Over a median follow-up of 11.4 years, 119 (19.8%) of the 601 patients died from a CV cause. In multivariable Cox proportional hazards regression models, adjusting for conventional risk factors, plasma citrate was associated with an increased risk of CV mortality (the hazard ratio (HR) per 1-SD increment was 1.19 (95%CI: 1.00–1.40), *p* = 0.048). This association was prominent in males (*n* = 49 with CV mortality) (HR 1.52 (95%CI: 1.14–2.03), *p* = 0.005), but not in females (*n* = 70 with CV mortality) (HR 1.11 (95%CI: 0.90–1.37), *p* = 0.319) (age-adjusted *P*_interaction_ = 0.044). In conclusion, higher plasma citrate levels are associated with an increased risk of CV mortality in patients with established T2D. Future studies are warranted to unravel the potential role of citrate-related pathways in the pathogenesis of T2D-related vascular complications.

## 1. Introduction

The enormous global burden of cardiovascular disease (CVD) in patients with Type 2 diabetes (T2D), the consequence of chronic hyperglycemia, and the reduced life expectancy of patients with this condition call for a better understanding of its underlying pathogenesis, aiming at introducing novel treatment targets [1,2,3,4,5]. The pathogenesis of T2D-associated CVD is multifactorial and involves many metabolic pathways, at least in part as a result of mitochondrial dysfunction [6,7,8,9]. Citrate is a key metabolite of the tricarboxylic acid (TCA) (Krebs or citric acid) cycle, a series of chemical reactions in the mitochondria that are responsible for the release of energy through the oxidation of acetyl-CoA derived from fats, proteins and carbohydrates, thereby forming a gateway for amphibolic metabolism [10,11,12].

Metabolites reflecting mitochondrial activity, in particular the circulating levels of citrate, have recently been suggested as potential biomarkers for cardiovascular and mortality risk assessments. Making use of a broad NMR metabolomic platform, which included 106 plasma lipid and non-lipid biomarkers, four biomarkers including citrate were identified as being prospectively associated with all-cause and cardiovascular (CV) mortality in the Estonian Biobank [13]. More recently, using data from the CATHeterization GENetics (CATHGEN) and Intermountain Heart Study high-CVD-risk cohorts, plasma citrate was chosen out of many possible lipids, lipoprotein parameters and metabolites to be part of a biomarker algorithm that predicts CV and all-cause mortality [14]. In the Avon Longitudinal Study of Parents and Children, citrate, as a metabolic trait, was found to be strongly associated with the risk of T2D, spanning from the age of 16 years until the onset of T2D several decades later [15]. Furthermore, cytosolic citrate, serving as a precursor for lipogenesis, has also been associated with the occurrence of kidney disease progression in T2D patients [16]. However, the extent to which plasma citrate may predict CV mortality in T2D patients treated in primary care is still unknown. 

Given that mitochondrial dysfunction is likely involved in the pathogenesis of diabetes-associated CV complications [10], we tested the hypothesis that plasma citrate levels, as an intermediate in the TCA cycle, could serve as a potential biomarker for the occurrence of CV mortality in individuals with T2D. Therefore, we investigated associations between plasma citrate, measured with NMR, and CV mortality in T2D patients enrolled in a primary-care-based prospective study in the Netherlands.

## 2. Materials and Methods

### 2.1. Study Population and Study Design

This study utilized samples and data from the ZODIAC cohort, which is a longitudinal observational cohort study conducted in the Zwolle region of the Netherlands. The study commenced in 1998 and focused on patients with established T2D who receive healthcare through primary care services. Our study was set up to investigate the potential utility of a shared-care project for patients with T2D, in which 61 general practitioners participated. Plasma samples obtained from participating individuals at baseline of the study were examined. Initially, at the start of the study, 1143 patients were enrolled. For the present study, patients with T2D without available follow-up data on their cardiovascular mortality and those without collected plasma samples available for citrate measurements were excluded. Following these exclusions, our final study population comprised 601 patients. The ZODIAC study was conducted in accordance with the Declaration of Helsinki guidelines. The study received ethical approval from the Institutional Review Board (IRB) of the Isala Hospital Zwolle, the Netherlands (IRB reference nos. 03.0316 and 07.0335), and all participants provided written informed consent before participating in the study.

### 2.2. Data Collection

Baseline data were collected during the annual check-up of patients by their general practitioner or nurse practitioner. Data included their medical history of CVD, tobacco consumption and medication use. Patients with a history of macrovascular complications, including angina pectoris, myocardial infarction, percutaneous transluminal coronary angioplasty, coronary artery bypass grafting, stroke, transient ischemic attack or peripheral vascular disease, were identified. During their visits to the outpatient clinic, their baseline measurements were taken. Their blood pressure was measured in supine position using a Welch Allyn sphygmomanometer after at least 5 min of rest. The participants’ height and weight were measured while they were standing without shoes and heavy outer garments. From these measurements, body mass index (BMI) was calculated by dividing body weight by height squared. Microvascular complications were defined based on specific criteria. Neuropathy was determined by assessing foot sensibility using a 5.07 Semmes–Weinstein monofilament test, with two or more errors indicating neuropathy in at least one foot. Diabetic retinopathy was evaluated using a retinal camera, and the assessment of fundus photos was conducted by an ophthalmologist. Nephropathy was defined as an estimated glomerular filtration rate (eGFR) below 60 mL/min/1.73 m^2^ and/or the presence of albuminuria, identified as an albumin-to-creatinine ratio exceeding 3.5 mg/mmol for women and 2.5 mg/mmol for men.

### 2.3. Study Outcomes

The primary endpoint of this study was CV mortality. In 2013, information regarding the patients’ vital status and cause of death was obtained from records kept by the hospital, general practitioners or the Municipal Personal Records Database. Causes of death were classified based on the International Classification of Diseases, Ninth Revision (ICD-9) coding system. CV death was specifically defined as a death whose principal cause was attributed to CV reasons, using ICD-9 codes ranging from 390 to 459 [17].

### 2.4. Laboratory Measurements

Citrate levels were determined in ethylenediaminetetraacetic acid (EDTA) anticoagulated plasma samples using nuclear magnetic resonance (NMR) spectroscopy at Labcorp (Morrisville, NC, USA). A detailed description of this methodology has been previously provided [18]. The stability of citrate in samples that were frozen at temperatures below −70 °C for up to 12 years has been established. Inter-assay precision for NMR-measured citrate exhibited coefficients of variation (%CV) ranging from 5.2% for a high-concentration pool to 9.6% for a low-concentration pool. Total cholesterol, HDL cholesterol and triglycerides and HbA1c were measured following routine laboratory methods as previously described [17].

### 2.5. Statistical Analysis

Baseline descriptive statistics of the study population were presented in two different formats. Continuous variables were expressed as means ± standard deviation (SD) or as medians with interquartile ranges (IQR). Categorical variables were presented as numbers along with their corresponding percentages (%). Baseline characteristics were stratified based on the future occurrence of cardiovascular mortality and on the presence or absence of macrovascular complications at baseline. To assess the differences in baseline characteristics between different groups (patients with and without macrovascular complications at baseline, and patients who died or not due to cardiovascular events during follow-up), statistical tests were conducted. Differences in continuous variables were analyzed by independent sample *t*-tests or Mann–Whitney *U*-tests where appropriate. Categorical variables were analyzed using chi-square tests or Fisher’s exact tests, as appropriate. Kaplan–Meier survival analysis was performed to assess survival distributions across tertiles of plasma citrate levels, which were compared using log-rank tests. Survival time was defined from baseline until the date of the last examination that participants attended, the date of their cardiovascular death, or 2012 (last year of follow-up as census date). Univariable and multivariable Cox proportional hazards regression analyses were performed to investigate the associations between lipoprotein particles and the risk of developing microvascular complications. Multivariable Cox proportional hazards regression models were constructed by step-wise addition of potentially confounding factors which included age and sex (*Model 2*), disease duration, the presence of macrovascular complications and HbA1c levels (Model 3), statin use (*Model 4*), smoking, the use of anti-hypertensive drugs and systolic blood pressure (*Model 5*). The results of Cox regression analyses were reported as hazard ratios (HRs) with corresponding 95% confidence intervals (CIs). Plasma citrate levels were standardized, and the HRs were expressed in 1-SD increments. The proportionality of hazards assumption was checked to ensure that it was not violated. Statistical analysis was conducted using SPSS Statistics 28.0 software (SPSS Inc., Chicago, IL, USA). A significance level of *p* < 0.05 (two-tailed) was considered statistically significant.

## 3. Results

### 3.1. Study Cohort Characteristics

Baseline characteristics were first compared between the patients who had macrovascular complications at baseline and those who did not (Table 1). The patients with macrovascular complications at baseline, i.e., angina pectoris, myocardial infarction, coronary artery bypass grafting, percutaneous coronary intervention, stroke, transient ischemia attack or peripheral vascular disease, died more frequently during the follow-up as compared to the patients without macrovascular complications (29.1% vs. 14.3%, *p* < 0.001). The patients with macrovascular complications at baseline were older, were more often male, had a lower diastolic blood pressure, more often used statins and antihypertensive drugs, had lower levels of total cholesterol and HDL cholesterol, and had lower renal function. Plasma citrate levels did not significantly differ between the patients with and without macrovascular complications at baseline. All the patients received diet and lifestyle advice and glucose-lowering medication that consisted of metformin, sulfonylurea and insulin (alone or in combination). Other glucose-lowering drugs were not used. Diabetes medication use was not significantly different between the groups.

### 3.2. Plasma Citrate Levels and the Risk of Cardiovascular Mortality

During a median follow-up of 11.4 (IQR: 7.5–14.3) years, 119 of the 601 patients (19.8%) (49 males (8.2%) and 70 females (11.6%)) died from a CV event. Of these, 23 patient (19.3%) deaths were due to ischemic heart disease, 35 to a stroke (29.4%), and 61 (51.3%) to an unspecified CV condition. Table 2 shows that the patients who died because of CV events were older, had a longer diabetes duration, more frequently had a history of macrovascular and microvascular complications, had higher HbA1c levels and had lower renal function. Glucose-lowering medication and statin use were not significantly different between the patients who died from CV causes compared to those who did not. The levels of total cholesterol, HDL cholesterol and triglycerides were not significantly different between the groups. Plasma citrate levels were higher in those who died from CV events during follow-up compared to patients who survived (*p* < 0.001). In the whole cohort (*n* = 601), the plasma citrate levels were higher in females (median 128 (IQR: 112–150) µmol/L) compared to males (median 119 (IQR: 100–138) µmol/L, *p* < 0.001).

**Figure 1:**
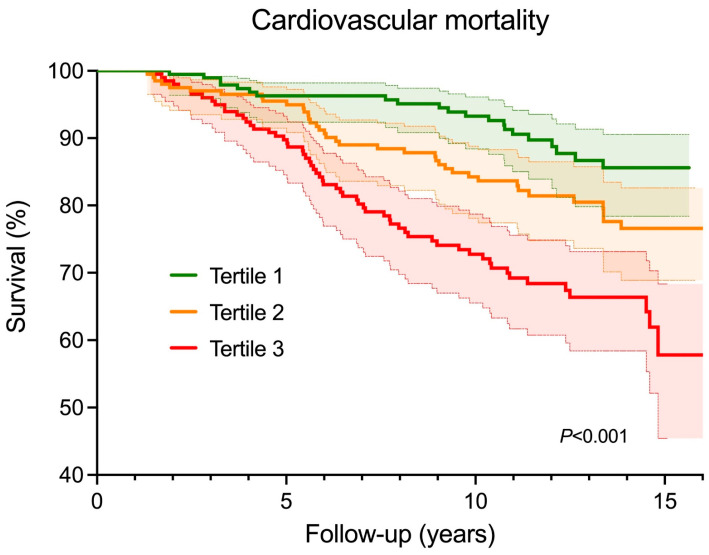
Kaplan–Meier survival curves for cardiovascular mortality according to tertiles of plasma citrate levels (tertile 1: <110 µmol/L; tertile 2: 110–136 µmol/L; tertile 3 > 136 µmol/L) (log-rank test, *p* < 0.001). Shaded areas indicate 95% confidence intervals.

The Kaplan–Meier survival analysis demonstrated statistically significant differences in survival distributions according to the tertiles of the plasma citrate levels (Figure 1, log-rank test, *p* < 0.001).

In the Cox proportional hazards regression analyses, the plasma citrate levels were associated with the risk of CV mortality (Table 3; A, *Model 1*, HR per 1-SD increment in plasma citrate levels was 1.48 (95% CI: 1.29–1.70), *p* < 0.001). After adjusting for age, sex, disease duration, HbA1c levels and the presence of macrovascular complications, this association remained (Table 3; A, *Model 3*, HR per 1-SD increment was 1.19 (95% CI: 1.01–1.40), *p* = 0.040). When additionally adjusting for statin use, anti-hypertensive medication, smoking and systolic blood pressure, the association between plasma citrate levels and the risk of CV mortality was still evident (*Model 5*, HR per 1-SD increment was 1.19 (95% CI: 1.00–1.41), *p* = 0.045).

In the age-adjusted analysis, there was an interaction between sex and plasma citrate levels on CV mortality (*P*_interaction_ = 0.044). Therefore, we performed sex-stratified Cox proportional hazards regression analyses (Table 3; B for males, Table 3; C for females). There was a stronger association between plasma citrate levels and the risk of CV mortality in males compared to females, whose results were only significant in the crude analysis. In males, plasma citrate levels were significantly associated with risk of CV mortality after adjusting for all covariates, both as continuous variables and as categorical variables.

In a secondary analysis, the association of citrate with CV mortality was determined after the exclusion of participants who died within two years of follow-up. The association remained in the fully adjusted model (*Model 5*, HR per 1-SD increment was 1.23 (95% CI: 1.03–1.46), *p* = 0.021) (Table 4; A). When analyzing the tertiles of the plasma citrate levels as an independent variable, the association between the highest citrate tertile remained associated with CV mortality when adjusted for age, sex, disease duration, HbA1c levels, the presence of macrovascular complications at baseline and statin use (*Model 4*, T3 vs. T1, HR per 1-SD increment was 1.69 (95% CI:1.00–2.88), *p* = 0.015). Furthermore, the sex difference remained but only in the crude analysis (*P*_interaction_ = 0.034) (Table 4; B and C).

## 4. Discussion

We demonstrated that within a primary-care-based cohort of patients with established T2D, higher circulating citrate concentrations are associated with an increased risk of CV mortality, even after adjusting for established risk factors. This association varied between the sexes, being present only in males. Collectively, our results suggest a potential pathogenic involvement of citrate-related pathways, reflected by plasma citrate levels, in the occurrence of CV death in patients with established T2D.

Mitochondrial dysfunction coincides with impaired fatty acid oxidation which contributes to oxidative stress in individuals with T2D [9,19]. In particular, metabolic changes in T2D involve an increased uptake of free fatty acids alongside the partial uncoupling of the mitochondrial electron transport chain. This results in the overproduction of free radicals, which culminates in oxidative stress. In line with this, serum free thiols as a proxy of oxidative stress defense were inversely associated with incidental CVD, as well as in subjects with T2D [20]. The concept of TCA abnormalities as a pathogenic mechanism contributing to T2D development and a risk of complications has gained further support from clinical observations in humans demonstrating elevated levels of ketone bodies to predict new onset T2D and to be associated with glycemic control [21,22]. Furthermore, the observed association between citrate levels and the risk of cardiovascular mortality in T2D patients could also be mediated in the gut microbiome, whose altered composition and functionality have been intricately associated with both T2D and the development of CVD [23]. For example, trimethylamine-N-oxide (TMAO), a liver-derived by-product of microbial metabolism, has been implicated in the pathogenesis of CVD [24] and was also found to be significantly associated with an increased risk of cardiovascular mortality in the ZODIAC cohort [25].

An independent association of plasma citrate with CV mortality has been reported in two large-scale prospective studies [13,14]. Another prospective study examined the associations between TCA cycle components, including citrate, and the risk of developing CV events and mortality after an acute coronary syndrome [26]. This study in high-risk individuals identified positive associations of CV outcome with isocitrate, aconitate, isocitrate, d/l-2-hydroxyglutarate and adverse outcomes but not with citrate. To our knowledge, our finding of a robust association of citrate with CV mortality in a T2D population, in particular in men, has not been documented before. 

Plasma citrate levels are higher in females than in males [11,27], as confirmed here. A striking observation of our study is that the association of citrate with CV mortality was pertinent in males only. Such a sexual dimorphism has also been found recently in a study showing increased plasma citrate levels with more advanced stages of liver cirrhosis [27], but the responsible mechanisms remain poorly understood. In male but not female rats, mitochondrial citrate synthase declines with age and is affected by diet [28]. Another large study identified sex differences in mitochondrial function, with male mice showing reduced function compared to female mice, which could be explained by their genetic make-up [29]. Another study in aging monkeys also demonstrated sex differences in mitochondrial metabolic pathways in cardiac tissue [30]. In humans with obesity, sex differences were identified for lipid compounds involved in the coupling of mitochondrial fatty acid transport, β-oxidation and TCA cycle flux [31]. 

The strengths of this study include the well-documented nature of this cohort, the large sample size and its prospective design. Since the association between plasma citrate levels and CV mortality remained present after excluding patients who died within two years of follow-up, the risk of reverse causation seems unlikely. Our study’s limitations also warrant recognition. This cohort consisted of primarily Dutch patients who were White, which could limit the generalizability of our findings. Also, the descriptive nature of our study precludes us from inferring causality. In other words, we were unable to pinpoint the exact mechanism underlying the observed association between plasma citrate levels and the risk of CV mortality in patients with T2D. Instead, we can only speculate about impaired mitochondrial functioning as a potential factor contributing to this. Furthermore, we lacked data on some potentially relevant confounders in this study, e.g., the habitual dietary intake of patients. Finally, since this cohort was primarily followed in the first decade of the 21st century, novel glucose-lowering medications, like GLP-1 receptor agonists or SGLT2 inhibitors, were not in use yet, as these drugs were not available in the time frame of the current study.

In conclusion, plasma citrate levels, as a proxy for TCA or citric acid cycle disturbances and mitochondrial dysfunction, are associated with an increased risk of CV mortality in patients with established T2D. Future research is required to understand the mechanistic underpinnings of this association.

## Figures and Tables

**Table 1:** Baseline characteristics of the study population divided by the presence or absence of macrovascular complications at baseline.

	Macrovascular Complications at Baseline (*n* = 223)	No Macrovascular Complications at Baseline (*n* = 378)	*p*-Value
Age (years)	70.0 ± 9.5	66.0 ± 11.4	<0.001
Sex			<0.001
Male, *n* (%)	117 (52.5)	137 (36.2)	
Female, *n* (%)	106 (47.5)	241 (63.8)	
BMI (kg/m^2^)	28.2 (25.2–31.6)	28.7 (26.0–31.2)	0.464
Disease duration (years)	5 (2–11)	4 (2–9)	0.062
CV mortality during follow-up, *n* (%)	65 (29.1)	54 (14.3)	<0.001
Systolic blood pressure (mmHg)	150 (135–170)	150 (140–170)	0.078
Diastolic blood pressure (mmHg)	80 (75–90)	85 (80–90)	<0.001
Current smoking, *n* (%)	40 (18.0)	63 (16.8)	0.703
HbA1c (%)	7.2 (6.3–8.2)	7.1 (6.3–8.2)	0.596
HbA1c (mmol/mol)	55.2 (46.5–65.0)	54.1 (45.4–66.1)	0.596
eGFR (ml/min/1.73 m^2^)	65.7 (53.0–81.0)	71.0 (58.0–91.1)	0.003
Diabetes treatment at baseline			0.119
Diet alone, *n* (%)	16 (14.3)	33 (16.9)	
Metformin, *n* (%)	43 (24.2)	71 (22.3)	
SU derivatives, *n* (%)	91 (51.1)	180 (56.6)	
Insulin, *n* (%)	32 (18.0)	40 (12.6)	
Both (insulin + oral antidiabetic), *n* (%)	7 (6.3)	15 (7.7)	
Statin use, *n* (%)	41 (18.6)	28 (7.4)	<0.001
Anti-hypertensive drugs, *n* (%)	137 (61.7)	147 (39.0)	<0.001
Total cholesterol (mmol/L)	5.30 (4.70–6.10)	5.60 (4.90–6.40)	0.031
HDL cholesterol (mmol/L)	1.09 (0.92–1.29)	1.13 (0.94–1.40)	0.016
Triglycerides (mmol/L)	2.18 (1.53–3.27)	2.15 (1.56–3.21)	0.654
Plasma citrate (µmol/L)	124 (110–147)	123 (104–143)	0.139

Data are presented as mean ± SD, median (IQR) or as proportions (*n*) with corresponding percentages (%). Abbreviations: BMI, body mass index; CV, cardiovascular; eGFR, estimated glomerular filtration rate; HbA1c; hemoglobin A1c; HDL, high-density lipoprotein; and SU, sulfonylurea.

**Table 2:** Baseline characteristics of the study population divided by the occurrence of cardiovascular (CV) mortality during follow-up.

	CV Mortality during Follow-Up (*n* = 119)	No CV Mortality during Follow-Up (*n* = 482)	*p*-Value
Age (years)	74.1 ± 7.9	65.9 ± 10.9	<0.001
Sex			0.789
Male, *n* (%)	49 (41.2)	205 (42.5)	
Female, *n* (%)	70 (58.8)	277 (57.5)	
BMI (kg/m^2^)	28.7 (25.7–31.1)	28.4 (25.7–31.5)	0.982
Disease duration (years)	6 (3–14)	4 (2–8)	<0.001
Systolic blood pressure (mmHg)	155 (140–170)	150 (140–170)	0.076
Diastolic blood pressure (mmHg)	80 (75–90)	80 (80–90)	0.187
Current smoking, *n* (%)	22 (18.6)	81 (16.9)	0.655
History of macrovascular complications, *n* (%)	65 (54.6)	158 (32.8)	<0.001
History of microvascular complications, *n* (%)	21 (70.0)	112 (45.0)	0.010
HbA1c (%)	7.3 (6.5–8.3)	7.0 (6.3–8.1)	0.026
HbA1c (mmol/mol)	56.3 (47.5–67.2)	53.0 (45.4–65.0)	0.026
eGFR (ml/min/1.73 m^2^)	59.0 (46.5–71.3)	72.4 (59.0–92.0)	<0.001
Diabetes treatment at baseline			0.862
Diet alone, *n* (%)	6 (13.6)	43 (16.3)	
Metformin, *n* (%)	33 (30.6)	81 (20.9)	
SU derivatives, *n* (%)	53 (49.1)	218 (56.2)	
Insulin, *n* (%)	20 (18.5)	52 (13.4)	
Both, *n* (%)	4 (9.1)	18 (6.8)	
Statin use, *n* (%)	11 (9.4)	58 (12.1)	0.416
Anti-hypertensive drugs, *n* (%)	60 (50.8)	224 (46.6)	0.404
Total cholesterol (mmol/L)	5.60 (4.80–6.30)	5.50 (4.80–6.30)	0.946
HDL cholesterol (mmol/L)	1.08 (0.88–1.30)	1.12 (0.94–1.36)	0.092
Triglycerides (mmol/L)	2.11 (1.46–2.97)	2.16 (1.58–3.25)	0.683
Plasma citrate (µmol/L)	138 (118–164)	120 (103–141)	<0.001

Data are presented as mean ± SD, median (IQR) or as proportions (*n*) with corresponding percentages (%). Abbreviations: BMI, body mass index; CV, cardiovascular; eGFR, estimated glomerular filtration rate; HbA1c; hemoglobin A1c; HDL, high-density lipoprotein; and SU, sulfonylurea.

**Table 3:** Cox proportional hazards regression analyses for associations between citrate concentrations and the risk of cardiovascular (CV) mortality in patients with type 2 diabetes.

A ALL	Citrate			T1	T2	T3
Model	HR	95% CI	*p*-value	<110 µmol/L	110–136 µmol/L	>136 µmol/L
1	1.48	1.29–1.70	**<0.001**	Reference	1.83 (1.08–3.13), ***p* = 0.026**	3.35 (2.04–5.51), ***p* < 0.001**
2	1.19	1.02–1.40	**0.029**	Reference	1.36 (0.80–2.32), *p* = 0.264	1.84 (1.10–3.07), ***p* = 0.021**
3	1.19	1.01–1.40	**0.040**	Reference	1.30 (0.76–2.23), *p* = 0.335	1.67 (1.00–2.79), ***p* = 0.048**
4	1.19	1.01–1.40	**0.040**	Reference	1.26 (0.73–2.16), *p* = 0.409	1.65 (0.99–2.77), *p* = 0.056
5	1.19	1.00–1.41	**0.045**	Reference	1.28 (0.74–2.20), *p* = 0.375	1.57 (0.94–2.64), *p* = 0.088
**B** **Males**	**Citrate**			**T1**	**T2**	**T3**
Model	HR	95% CI	*p*-value	<110 µmol/L	110–136 µmol/L	>136 µmol/L
1	1.83	1.47–2.27	**<0.001**	Reference	4.54 (1.81–11.4), ***p* = 0.001**	8.70 (3.55–21.3), ***p* < 0.001**
2	1.40	1.08–1.80	**0.010**	Reference	2.55 (1.00–6.49), ***p* = 0.049**	3.68 (1.44–9.41), ***p* = 0.006**
3	1.42	1.08–1.88	**0.013**	Reference	2.53 (0.99–6.48), *p* = 0.053	3.35 (1.31–8.62), ***p* = 0.012**
4	1.48	1.13–1.95	**0.005**	Reference	3.07 (1.15–8.18), ***p* = 0.025**	4.17 (1.54–11.3), ***p* = 0.005**
5	1.50	1.13–2.00	**0.006**	Reference	3.05 (1.14–8.20), ***p* = 0.027**	3.80 (1.39–10.4), ***p* = 0.009**
**C** **Females**	**Citrate**			**T1**	**T2**	**T3**
Model	HR	95% CI	*p*-value	<110 µmol/L	110–136 µmol/L	>136 µmol/L
1	1.32	1.08–1.60	**0.006**	Reference	0.94 (0.48–1.85), *p* = 0.851	1.78 (0.97–3.25), *p* = 0.062
2	1.10	0.89–1.34	0.378	Reference	0.89 (0.45–1.76), *p* = 0.747	1.22 (0.66–2.24), *p* = 0.525
3	1.12	0.91–1.37	0.280	Reference	0.90 (0.46–1.79), *p* = 0.771	1.25 (0.68–2.29), *p* = 0.447
4	1.12	0.91–1.37	0.288	Reference	0.87 (0.43–1.72), *p* = 0.679	1.23 (0.67–2.27), *p* = 0.502
5	1.12	0.91–1.39	0.287	Reference	0.89 (0.44–1.78), *p* = 0.736	1.21 (0.65–2.24), *p* = 0.550

HRs are expressed per 1-SD increment. Model 1: crude. Model 2: model 1, plus age and sex. Model 3: model 2, plus adjustment for disease duration, HbA1c and history of macrovascular complications. Model 4: model 3, with adjustment for statin use. Model 5: model 4, with adjustment for smoking, antihypertensive drugs and systolic blood pressure. Bold *p*-values indicate statistical significance.

**Table 4:** Cox proportional hazards regression analyses for associations between citrate concentrations and the risk of cardiovascular (CV) mortality in patients with type 2 diabetes, while excluding patients with less than two years of follow-up.

**A** **ALL**	**Citrate**			**T1**	**T2**	**T3**
Model	HR	95% CI	*p*-value	<110 µmol/L	110–136 µmol/L	>136 µmol/L
1	1.51	1.31–1.74	**<0.001**	Reference	1.68 (0.96–2.92), *p* = 0.068	3.39 (2.04–5.64), ***p* < 0.001**
2	1.23	1.04–1.44	**0.014**	Reference	1.24 (0.71–2.17), *p* = 0.447	1.88 (1.11–3.19), ***p* = 0.019**
3	1.22	1.03–1.44	**0.021**	Reference	1.20 (0.69–2.10), *p* = 0.525	1.71 (1.01–2.89), ***p* = 0.046**
4	1.22	1.03–1.44	**0.020**	Reference	1.16 (0.66–2.03), *p* = 0.615	1.69 (1.00–2.88), ***p* = 0.015**
5	1.23	1.03–1.46	**0.021**	Reference	1.19 (0.67–2.09), *p* = 0.552	1.61 (0.94–2.75), *p* = 0.081
**B** **Males**	**Citrate**			**T1**	**T2**	**T3**
Model	HR	95% CI	*p*-value	<110 µmol/L	110–136 µmol/L	>136 µmol/L
1	1.85	1.48–2.31	**<0.001**	Reference	4.09 (1.61–10.4), ***p* = 0.003**	8.17 (3.30–20.2), ***p* < 0.001**
2	1.42	1.10–1.84	**0.008**	Reference	2.33 (0.90–5.99), *p* = 0.081	3.50 (1.36–9.05), ***p* = 0.010**
3	1.43	1.08–1.91	**0.014**	Reference	2.32 (0.89–6.01), *p* = 0.085	3.09 (1.19–8.07), ***p* = 0.021**
4	1.50	1.13–2.00	**0.005**	Reference	2.85 (1.05–7.72), ***p* = 0.040**	3.91 (1.42–10.8), ***p* = 0.008**
5	1.53	1.14–2.06	**0.005**	Reference	2.84 (1.04–7.76), ***p* = 0.042**	3.48 (1.24–9.73), ***p* = 0.018**
**C** **Females**	**Citrate**			**T1**	**T2**	**T3**
Model	HR	95% CI	*p*-value	<110 µmol/L	110–136 µmol/L	>136 µmol/L
1	1.36	1.11–1.65	**0.003**	Reference	0.84 (0.41–1.73), *p* = 0.643	1.87 (1.00–3.47), ***p* = 0.049**
2	1.13	0.92–1.39	0.239	Reference	0.80 (0.39–1.64), *p* = 0.540	1.29 (0.69–2.41), *p* = 0.429
3	1.16	0.94–1.42	0.173	Reference	0.81 (0.39–1.66), *p* = 0.560	1.32 (0.70–2.47), *p* = 0.388
4	1.15	0.94–1.42	0.180	Reference	0.76 (0.37–1.58), *p* = 0.462	1.30 (0.69–2.44), *p* = 0.417
5	1.16	0.94–1.44	0.167	Reference	0.79 (0.38–1.64), *p* = 0.522	1.28 (0.68–2.42), *p* = 0.444

HRs are expressed in 1-SD increments. Model 1: crude. Model 2: model 1, plus age and sex. Model 3: model 2, plus adjustment for disease duration, HbA1c and history of macrovascular complications. Model 4: model 3, with adjustment for statin use. Model 5: model 4, with adjustment for smoking, antihypertensive drugs and systolic blood pressure. Bold *p*-values indicate statistical significance. Bold *p*-values indicate statistical significance.

## Data Availability

The datasets used and analyzed during the current study are available from the corresponding author on reasonable request.

## References

[1] Gæde P., Vedel P., Larsen N., Jensen G.V., Parving H.-H., Pedersen O. (2003). Multifactorial intervention and cardiovascular disease in patients with type 2 diabetes. N. Engl. J. Med..

[2] Wang C.C.L., Hess C.N., Hiatt W.R., Goldfine A.B. (2016). Clinical Update: Cardiovascular Disease in Diabetes Mellitus: Atherosclerotic Cardiovascular Disease and Heart Failure in Type 2 Diabetes Mellitus - Mechanisms, Management, and Clinical Considerations. Circulation.

[3] American Diabetes Association Professional Practice Committee 10 (2021). Cardiovascular Disease and Risk Management: Standards of Medical Care in Diabetes—2022. Diabetes Care.

[4] Davies M.J., D’Alessio D.A., Fradkin J., Kernan W.N., Mathieu C., Mingrone G., Rossing P., Tsapas A., Wexler D.J., Buse J.B. (2018). Management of Hyperglycemia in Type 2 Diabetes, 2018. A Consensus Report by the American Diabetes Association (ADA) and the European Association for the Study of Diabetes (EASD). Diabetes Care.

[5] Tomic D., I Morton J., Chen L., Salim A., Gregg E.W., E Pavkov M., Arffman M., Balicer R., Baviera M., Dam E.B.-V. (2022). Lifetime risk, life expectancy, and years of life lost to type 2 diabetes in 23 high-income jurisdictions: A multinational, population-based study. Lancet Diabetes Endocrinol..

[6] Poznyak A., Grechko A.V., Poggio P., Myasoedova V.A., Alfieri V., Orekhov A.N. (2020). The Diabetes Mellitus–Atherosclerosis Connection: The Role of Lipid and Glucose Metabolism and Chronic Inflammation. Int. J. Mol. Sci..

[7] Singh A., Kukreti R., Saso L., Kukreti S. (2022). Mechanistic Insight into Oxidative Stress-Triggered Signaling Pathways and Type 2 Diabetes. Molecules.

[8] Stratmann B. (2022). Dicarbonyl Stress in Diabetic Vascular Disease. Int. J. Mol. Sci..

[9] La Sala L., Prattichizzo F., Ceriello A. (2019). The link between diabetes and atherosclerosis. Eur. J. Prev. Cardiol..

[10] Gaster M., Nehlin J.O., Minet A.D. (2012). Impaired TCA cycle flux in mitochondria in skeletal muscle from type 2 diabetic subjects: Marker or maker of the diabetic phenotype?. Arch. Physiol. Biochem..

[11] Pinti M.V., Fink G.K., Hathaway Q.A., Durr A.J., Kunovac A., Hollander J.M. (2019). Mitochondrial dysfunction in type 2 diabetes mellitus: An organ-based analysis. Am. J. Physiol. Endocrinol. Metab..

[12] Costello L.C., Franklin R.B. (2016). Plasma Citrate Homeostasis: How It Is Regulated; And Its Physiological and Clinical Implications. An Important, But Neglected, Relationship in Medicine. HSOA J. Hum. Endocrinol..

[13] Fischer K., Kettunen J., Würtz P., Haller T., Havulinna A.S., Kangas A.J., Soininen P., Esko T., Tammesoo M.-L., Mägi R. (2014). Biomarker profiling by nuclear magnetic resonance spectroscopy for the prediction of all-cause mortality: An observational study of 17,345 persons. PLOS Med..

[14] Otvos J.D., Shalaurova I., May H.T., Muhlestein J.B., Wilkins J.T., McGarrah R.W., E Kraus W. (2023). Multimarkers of metabolic malnutrition and inflammation and their association with mortality risk in cardiac catheterisation patients: A prospective, longitudinal, observational, cohort study. Lancet Healthy Longev..

[15] Bell J.A., Bull C.J., Gunter M.J., Carslake D., Mahajan A., Smith G.D., Timpson N.J., Vincent E.E. (2020). Early Metabolic Features of Genetic Liability to Type 2 Diabetes: Cohort Study With Repeated Metabolomics Across Early Life. Diabetes Care.

[16] Afshinnia F., Nair V., Lin J., Rajendiran T.M., Soni T., Byun J., Sharma K., Fort P.E., Gardner T.W., Looker H.C. (2019). Increased lipogenesis and impaired β-oxidation predict type 2 diabetic kidney disease progression in American Indians. JCI Insight.

[17] Riphagen I.J., Boertien W.E., Alkhalaf A., Kleefstra N., Gansevoort R.T., Groenier K.H., Van Hateren K.J., Struck J., Navis G., Bilo H.J. (2013). Copeptin, a surrogate marker for ar-ginine vasopressin, is associated with cardiovascular and all-cause mortality in patients with type 2 diabetes (ZODIAC-31). Diabetes Care.

[18] Garcia E., Connelly M.A., Matyus S.P., Otvos J.D., Shalaurova I. (2021). High-throughput nuclear magnetic resonance measurement of citrate in serum and plasma in the clinical laboratory. Pract. Lab. Med..

[19] Sivitz W.I., Yorek M.A. (2010). Mitochondrial dysfunction in diabetes: From molecular mechanisms to functional significance and therapeutic opportunities. Antioxid. Redox Signal..

[20] Abdulle A.E., Bourgonje A.R., Kieneker L.M., Koning A.M., Gemert S.l.B.-V., Bulthuis M.L.C., Dijkstra G., Faber K.N., Dullaart R.P.F., Bakker S.J.L. (2020). Serum free thiols predict cardiovascular events and all-cause mortality in the general population: A prospective cohort study. BMC Med..

[21] Szili-Torok T., de Borst M.H., Garcia E., Gansevoort R.T., Dullaart R.P., Connelly M.A., Bakker S.J.L., Tietge U.J.F. (2023). Fasting ketone bodies and incident type 2 diabetes in the general population. Diabetes.

[22] van der Vaart A., Knol M.G.E., de Borst M.H., Bakker S.J.L., Connelly M.A., Garcia E., Bilo H.J.G., van Dijk P.R., Dullaart R.P.F. (2022). The Paradoxical Role of Circulating Ketone Bodies in Glycemic Control of Individuals with Type 2 Diabetes: High Risk, High Reward?. Biomolecules.

[23] Sikalidis A.K., Maykish A. (2020). The Gut Microbiome and Type 2 Diabetes Mellitus: Discussing A Complex Relationship. Biomedicines.

[24] Tang W.H., Wang Z., Levison B.S., Koeth R.A., Britt E.B., Fu X., Wu Y., Hazen S.L. (2013). Intestinal microbial metabolism of phosphatidylcholine and cardiovascular risk. N. Engl. J. Med..

[25] Flores-Guerrero J.L., van Dijk P.R., Connelly M.A., Garcia E., Bilo H.J.G., Navis G., Bakker S.J.L., Dullaart R.P.F. (2021). Circulating Trimethyl-amine N-Oxide Is Associated with Increased Risk of Cardiovascular Mortality in Type-2 Diabetes: Results from a Dutch Diabetes Cohort (ZODIAC-59). J. Clin. Med..

[26] Sanchez-Gimenez R., Peiró M., Bonet G., Carrasquer A., Fragkiadakis G.A., Bulló M., Papandreou C., Bardaji A. (2023). TCA cycle metabolites associated with adverse outcomes after acute coronary syndrome: Mediating effect of renal function. Front. Cardiovasc. Med..

[27] Amjad W., Shalaurova I., Garcia E., Gruppen E.G., Dullaart R.P.F., DePaoli A.M., Jiang Z.G., Lai M., Connelly M.A. (2023). Circulating Citrate Is Associated with Liver Fibrosis in Nonalcoholic Fatty Liver Disease and Nonalcoholic Steatohepatitis. Biomolecules.

[28] Schneider J., Han W.H., Matthew R., Sauvé Y., Lemieux H. (2020). Age and sex as confounding factors in the relationship between cardiac mitochondrial function and type 2 diabetes in the Nile Grass rat. PLoS ONE.

[29] Norheim F., Hasin-Brumshtein Y., Vergnes L., Krishnan K.C., Pan C., Seldin M.M., Hui S.T., Mehrabian M., Zhou Z., Gupta S. (2019). Gene-by-Sex Interactions in Mitochondrial Functions and Cardio-Metabolic Traits. Cell Metab..

[30] Yan L., Ge H., Li H., Lieber S., Natividad F., Resuello R., Kim S.-J., Akeju S., Sun A., Loo K. (2004). Gender-specific proteomic alterations in glycolytic and mitochondrial pathways in aging monkey hearts. J. Mol. Cell. Cardiol..

[31] Broussard J.L., Perreault L., Macias E., Newsom S.A., Harrison K., Bui H.H., Milligan P., Roth K.D., Nemkov T., D’Alessandro A. (2021). Sex Differences in Insulin Sensitivity are Related to Muscle Tissue Acylcarnitine But Not Subcellular Lipid Distribution. Obes. (Silver Spring).

